# Toward an evolutionary account of the changes in the human pitch vocal system

**DOI:** 10.3389/fpsyg.2023.1249727

**Published:** 2023-10-19

**Authors:** Antonio Benítez-Burraco

**Affiliations:** Department of Spanish, Linguistics, and Theory of Literature (Linguistics), Faculty of Philology, University of Seville, Seville, Spain

**Keywords:** pitch, self-domestication, speech, *ABCC9*, vocal behavior

## Introduction

Language is probably the most distinctive of human traits. We are endowed with a species-specific capacity for generating complex arrays of symbols that encode equally complex thoughts, which can be in turn shared with others to fulfill different purposes: informing, manipulating, socializing, amusing, and many others. Language is usually externalized via speech, although deaf people use gestures for conveying linguistic meanings. How language evolved has been a serious concern for many disciplines, from linguistics to anthropology to human history. Specifically, it is not clear when we started to use sounds for sharing our linguistic thoughts. Because apes rely on gestures for voluntary information exchanges (Graham et al., 2022; Hobaiter et al., 2022), it has been argued that language evolved “from hand to mouth” (Corballis, 2002) as a result of some brain rewiring (Brown et al., 2021), also because the primate vocal tract is essentially speech-ready (Fitch et al., 2016). Nonetheless, comparative and paleoanthropological research suggests that some minor anatomical modifications in our speech organs, seemingly associated to the evolution of human-like languages, have occurred in the human lineage (Lieberman and McCarthy, 1975; Blasi et al., 2019; Dediu et al., 2021; Nishimura et al., 2022). In turn, we can expect that some of the changes experienced by our vocal system resulted from genetic and epigenetic changes. This possibility is supported by the existence of several genetic conditions impacting on our speech abilities (Sataloff, 1995; Newbury and Monaco, 2010; Morgan, 2013), but also by the finding that the regions that are differentially methylated in modern humans compared to Neanderthals and Denisovans are enriched in genes associated with face and vocal tract anatomy (Gokhman et al., 2020). Nonetheless, we still lack a good understanding of how and why selected genetic and epigenetic changes impacted on specific speech features.

## Genetics of the human pitch vocal system

Gisladottir et al. (2023) have recently uncovered several common variants in the gene *ABCC9* that are associated with higher voice pitch. In addition, these variants are associated with reduced expression of *ABCC9* in the adrenal glands and greater pulse pressure, implicating hormonal and cardiovascular systems. Finally, according to Open Target Genetics (https://genetics.opentargets.org/), the same variants are associated with the expression of the gene in the dorsolateral prefrontal cortex in the Common Mind dataset. This brain region has experienced molecular, cellular, and structural changes in the human lineage (Falk, 2014; Ma et al., 2022), including changes in the expression pattern of *FOXP2* (Ma et al., 2022), a gene linked to speech abilities (Morgan et al., 2016). Interestingly as well, most of the common variants associated with higher voice pitch uncovered by Gisladottir et al. (2023) are derived compared to apes. These authors also identified a fixed change in the coding region of the gene that occurred after our split from great apes, but before the split between modern humans and Neanderthals/Denisovans. Lastly, another region of the gene is among the set of accelerated regions in humans compared to other primates (Bi et al., 2023). All this evidence suggests that the *ABCC9* gene might have been subject to positive selection in our lineage, with some potential impact, specifically, on our speech abilities, the control of stress, and aspects of brain function.

Although the distinctive attributes of human speech mostly depend on its segmental components (as vowels or consonants), suprasegmental features are also important. These include pitch, which is the perceptive quality of sound frequency. Tonal languages use changes in pitch levels and contours to convey different lexical and grammatical meanings. More generally, pitch is one key prosodic cue, which is used for marking the main structural components of an utterance (like phrases or clauses) or sentence types (like affirmative vs. interrogative). Gisladottir et al. (2023) finding is a valuable contribution to the ongoing efforts for decoding the genetics and the evolution of the human vocal system, as it provides a direct link between one specific component of human speech and one specific gene. This commentary paper is aimed to hypothesize about one possible rationale for the selection events on this gene, as part of the evolution of human language and the human species, more generally. Nonetheless, other alternative explanations for the observed genetic variations and their relationship to voice pitch are conceivable, given the multiple functions performed by *ABCC9*, particularly at the brain level (see Nelson et al., 2015 for a review).

## A rationale for the genetic changes in *ABCC9* impacting on the human pitch vocal system

Interestingly enough, the gene highlighted by Gisladottir et al. (2023) is positively selected in tamed foxes (Trut et al., 2009), which have been used as an animal model of domestication processes (Trut et al., 2009; but see Lord et al., 2020 for a critical view). Domestication usually gives rise to a distinctive set of changes in different parts of the body, the so-called “domestication syndrome” (Wilkins et al., 2014; see Sánchez-Villagra et al., 2016 for some criticism). These include modifications in the skull, the facial area, and the vocal tract (Riede and Fitch, 1999; Kruska, 2005; Trut et al., 2009; Zeder, 2012; Wilkins et al., 2014). Also, changes in vocalization patterns are commonly observed in domesticated animals, typically resulting in more complex and varied vocalizations (Corbett, 2004; Okanoya, 2017). Specifically, domestic animals can produce vocalizations with a higher pitch frequency compared to their wild conspecifics, as observed in cats (Nicastro, 2004; Yeon et al., 2011). It has been argued that these coordinated modifications of the body of domesticates result, at least in part, from changes in the hypothalamic-pituitary adrenal (HPA) axis in response to selection for tameness and increased tolerance to humans, which is usually the first step in any domestication process (Wilkins et al., 2014). Significantly, the *ABCC9* variants uncovered by Gisladottir et al. (2023) are also associated, as noted, with the expression of the gene in the adrenal glands. Overall, this evidence suggests that *ABCC9* might be part of the genetic architecture of domestication.

All this might be relevant for human evolution, and particularly, the evolution of speech, if one considers some accounts of the human history according to which our species experienced a process similar to animal domestication. This view is commonly referred to as the human self-domestication (HSD) hypothesis (Hare, 2017; Wrangham, 2019). In brief, it is argued, diverse external factors (living in harsher climatic conditions, the colonization of new territories, the advent of co-parenting) might have promoted a selection toward increased prosocial behavior in the hominin group and ultimately, have triggered the physiological mechanisms underlying domestication events, as sketched above, including changes in the HPA axis. In turn, this is expected to have resulted in a constellation of body, cognitive, and behavioral changes in humans that parallel domestication features in other species. In fact, the external factors that seemingly favored prosocial behaviors in humans can be expected to trigger self-domestication features in any species, as observed in bonobos (Hare et al., 2012) or elephants (Raviv et al., 2023). In the case of humans, the traits resulting from our self-domestication include modifications in the skull and the facial area that have been characterized as an increased “feminization” of inherited hominin features. These changes ultimately entail a reduction of sexual dimorphic traits and a potentiation of neotenic (i.e., childish) features. Interestingly, signs of this “feminization” appear variably during our history, with a peak during the Upper Paleolithic (Cieri et al., 2014), when modern languages, endowed with more complex phonologies and demanding more sophisticated speech abilities, have been claimed to have emerged (Benítez-Burraco and Progovac, 2020). Signs of “feminization,” and ultimately of HSD, also appear variably in present-day populations, arguably in response to differences in social conditions and behavior, including the status of women in society (Gleeson and Kushnick, 2018). A higher pitch voice could be considered a more feminine trait and accordingly, a less threatening, more self-domesticated phenotype, contrary to a lower pitch, associated to male rudeness (Aung and Puts, 2020). Levinson (2022) has discussed these high-pitched vocalizations in relation to “cuteness selection.” In essence, this process involves the generalization of mother–infant interaction patterns to all adults and ultimately, the spread of the sort of interactions that promote the acquisition of culturally transmitted systems like human languages (see Benítez-Burraco and Kempe, 2018 for discussion). It happens that mother-infant interactions also feature a higher pitch in other social species, like bottlenose dolphins (Sayigh et al., 2023). More generally, changes in vocal behavior have been observed in other primates claimed to have gone through a self-domestication process, including bonobos (Gruber and Clay, 2016), and marmoset monkeys, in which increased affiliative vocal behaviors correlate with the size of facial fur patches, a hallmark of self-domestication (Ghazanfar et al., 2020).

In our view, and summarizing the discussion above, HSD might have accelerated changes in *ABCC9* due to its role in the human vocal repertoire and other characteristics relevant for the HSD phenotype. More specifically, we wish to argue that HSD could have resulted in selected changes in *ABCC9* that favored higher pitch vocal sounds, which were later selected, or co-opted, as part of a general trend toward increased cuteness. Ultimately, these changes might have contributed to the potentiation of cultural mechanisms (e.g., bonding systems) that promote the complexification and diversification of languages. This possibility is supported by the phenotypic profile of clinical conditions resulting from mutations in *ABCC9* in present-day populations. One of these conditions is Cantú Syndrome (OMIM#239850), which presents with an attenuation of most of the traits impacted by (self-)domestication, including macrocephaly, increased body size, hypertrichosis, coarse facial features, and significantly, a hoarse voice which is suggestive of a lower pitch (Grange et al., 2014). With regards to the cognitive profile and social functioning abilities of the affected people, they show symptoms of autism spectrum disorder (ASD), particularly, at younger ages (Roessler et al., 2021). ASD has been argued to entail reduced features of HSD too (Benítez-Burraco et al., 2016). Needless to say, because of the pleiotropic nature of genes, one could imagine other explanations for the selection of the genetic variants attested by Gisladottir et al. (2023). For properly testing the hypothesis sketched in this commentary, further *in silico* and *in vitro* analyses are needed, including a survey of these variants in a larger number of domestic species. More specifically, generating animal models bearing some of the most promising variants could enable to uncover the true mechanism accounting for the changes observed in (and hypothesized for) voice pitch.

## Discussion

To conclude, the research by Gisladottir et al. (2023) opens a promising avenue of research that could result in the formulation of more robust bridging hypotheses between the genetic changes occurred in the course of human evolution, the changes experienced by our body and behavior, and the emergence of human distinctive traits, specifically, our idiosyncratic speech abilities.

## Author contributions

AB-B conceived the paper, reviewed the available literature, analyzed the data, and wrote the paper.
